# The Loss of Children

**Published:** 1882-07

**Authors:** 


**The Loss of Children.**—Those who have never passed through this fiery furnace, which tries the inmost heart, can not sympathize with bereaved parents whose hearts bleed over their children dead. To describe the anguish which rends their hearts, as they gaze upon the loved forms on whom their fondest hopes and highest aspirations had rested so firmly, now cold and lifeless in their coffin-home, would require a pen dipped in the very essence of the sublimest sorrow itself. None but the parents can feel it, and none but those who have mourned like them, can sympathize with those who mourn the death of their children. The loss no power on earth can make good or even alleviate. No power on earth can bring them back, and place them again beneath their parents' loving gaze and fond care. From earth they have taken their final departure, never, never to return. The little chair they occupied, the little plate and the knife and fork they used, will be to them of service no more—but merely lonely mementoes of their existence. The patter of their little feet upon the floor, and the music of their sweet, sweet voices, will greet the parents' ear never again on earth. All will be a recurrence of all that is dreary and dismal. But hope, plumed by religion, points to a happy meeting in another and better world.

**Secret of Eloquence.**—I owe my success in life to one single fact, namely: At the age of twenty-seven I commenced, and continued for years, the process of daily reading and speaking upon the contents of some historical or scientific book. These off-hand efforts were made sometimes in a corn-field, at others in the forest, and not unfrequently in some distant barn, with the horse and cow for my auditors. It is to this early practice in the great art of all arts that I am indebted for the primary and leading impulses that stimulated me forward, and shaped and molded my entire subsequent destiny. Improve, then, young gentlemen, the superior advantages you here enjoy. Let not a day pass without exercising your powers of speech. There is no power like that of oratory. Cæsar controlled men by exciting their fears; Cicero, by captivating their affections, and swaying their passions. The influence of the one perished with its author; that of the other continues to this day.—HENRY CLAY.

**Christianity Aggressive.**—The defensive armor of a shrinking or timid policy does not suit her. Hers is then aked majesty of truth; and with all the grandeur of age, but with none of its infirmities, has she come down to us, and gathered strength from the battles she has fought and the victories she has won in the many conflicts of many generations. With such a religion as this, there is nothing to hide. All should be above board; and the broadest light of day should be made freely to circulate through all her secresies. But secrets she has none. To her belong the frankness and simplicity of conscious greatness; and whether she grapple it with pride of philosophy, or stand in front opposition to the prejudices of the multitude, she does it upon her own strength, and spurns all the props and all the auxiliaries of superstition away from

GRAHAM BREAD.—Take the unbolted flour of wheat, wet it with lukewarm water, add salt and yeast, knead in enough more of this flour to make it stiff, add a little molasses, and, when risen, bake in medium-sized loaves.

ERYSIPELAS is said to be cured by applying to the part affected, a paste made of raw cranberries beaten.

KETTLES are cleansed of onion and other odors, by dissolving a teaspoonful of pearlash or saleratus in water, and washing them.

HAIR, removed by fevers and other sickness, is made to grow by washing the scalp with a strong decoction of sage leaves once or twice a day.

STINGS and bites are often instantaneously cured by washing them in hartshorn or turpentine.

FLIES DESTROYED.—A pint of sweet milk, a quarter of a pound of sugar, two ounces of ground pepper, simmer together for ten minutes, and place it about in shallow dishes. If this is true, there is no necessity for using poisonous articles about a house.

BOILING POTATOES.—It is said that in Ireland they always nick off a piece of the skin, put them in a pot of cold water, which is gradually heated, but never allowed to boil; cold water should be added as soon as the water begins to boil; when done, pour all the water off, cover the vessel with a cloth, and in a few minutes they are cool enough for use.

BUY the best articles for family use; for, although they cost more, good articles spend best.

SUGAR from Havana is always dirty, that from Brazil is clean, as also from Porto Rico and Santa Cruz. Refined sugars, whether loaf, crushed, or granulated, are the cheapest in the end.

LARD from a hog not over a year old, is the best, and should be hard and white.

BUTTER made in September and October is the best for winter use.

RICH cheese feels softer under the pressure of the finger. That which is very strong is neither very good nor healthy. To keep one that is cut, tie it up in a bag that will not admit flies, and hang it in a dry cool place. If mold appears on it, wipe it off with a dry cloth.

